# Assessing claims of counterproductivity of Just Stop Oil’s civil disobedience

**DOI:** 10.1038/s44168-026-00347-5

**Published:** 2026-02-27

**Authors:** Oscar Berglund, Colin J. Davis, Samuel Finnerty

**Affiliations:** 1 School for Policy Studies, University of Bristol, Bristol, UK; 2 School of Psychological Science, University of Bristol, Bristol, UK; 3 Department of Psychology, Lancaster University, Lancaster, UK

**Keywords:** Cultural and media studies, Cultural and media studies, Politics and international relations

## Abstract

A key development in the post-pandemic climate movement has been the rise of small-scale civil disobedience groups, who prioritise high-profile disruptions to gain media attention. The British group Just Stop Oil (JSO) have been one of the most publicised. In this paper we research the impact of JSO and ask if they have been counterproductive, as is often claimed. We make a significant contribution to the interdisciplinary literature on climate activism by proposing a two-dimensional framework for assessing counterproductivity. We conducted a rigorous mixed-methods study, analysing how JSO’s campaigning influenced media reporting and public opinion. This included media content analysis and public opinion surveys to evaluate how JSO’s protests shaped discourse on climate policy, public attitudes, and government responses. Our findings show that JSO achieved considerable media visibility, yet it has had limited success in broadening public awareness of climate change. However, they did contribute to increasing the salience of the issue of new oil and gas licences, compelling political actors to publicly address the matter. JSO have not been counterproductive in the sense that they have not turned public or policymakers against Net Zero policies. Their impact on support for the climate movement and state repression is more ambiguous.

## Introduction

The British climate activist group Just Stop Oil (JSO) announced in March 2025 that they would cease their disruptive protests, claiming that they had achieved their demand of banning new licences for oil and gas extraction in the North Sea. This was in the face of repeated accusations from commentators that their protests were counterproductive. JSO have been one of the more internationally prominent manifestations of small-scale civil disobedience, focusing on specific policy demands, such as banning fossil fuel exploration, retrofitting homes, or restoring wetlands. These groups, often linked through the A22 network, prioritise high-profile disruptions to gain media attention^1,2^. This strategy has amplified their visibility but also drawn significant controversy. Many have been partially funded by the US-based Climate Emergency Fund and rely on nonviolent action, though some have included minor acts of sabotage in their repertoires. Groups we classify as small-scale (in numbers of people) civil disobedience groups include JSO and Insulate Britain in the UK, Climate Defiance in the US, Last Generation in Europe, and Återställ Våtmarker in Sweden.

Much debate in public discourse and academic scholarship across disciplines surrounds the impact of these controversial high-profile protest tactics, with significant focus on whether or not they are counterproductive. Strong claims about counterproductivity have been made by prominent climate commmunicators^3^, and leading politicians^4^. Such claims have also found some support from experimental studies^5–7^. This paper makes a significant contribution to these debates by proposing a two-dimensional framework to conceptualise counterproductivity. We examine the impact of JSO and ask what impact they had on public opinion and public policy, and whether their three-year campaign was counterproductive. JSO were founded in 2022 and their primary demand was for ‘the UK Government [to] stop licensing all new oil, gas and coal projects’ ^8^. JSO perceive their role as ‘civil resistance…applying nonviolent pressure until [they] force change to happen’. The group has become well known in the UK for disrupting traffic, sporting events, cultural events, and museums.

JSO’s actions have frequently been framed as counterproductive by political commentators, the media, and sections of the public. This framing implies multiple interpretations: that their disruptions alienate potential supporters, shift support away from climate policy, make pro-environmental policy less likely to be enacted, or provide justification for government repression. This variance in interpretation requires unpacking to understand whether such tactics are counterproductive in terms of public attitudes toward the movement, support for specific policy demands, or the broader political consequences of protest actions. In this paper, we interrogate this counterproductive framing, assessing whether JSO succeeded in getting media attention, whether they were effective in communicating their message, and whether they shifted public opinion on relevant issues.

**Fig. 1:**
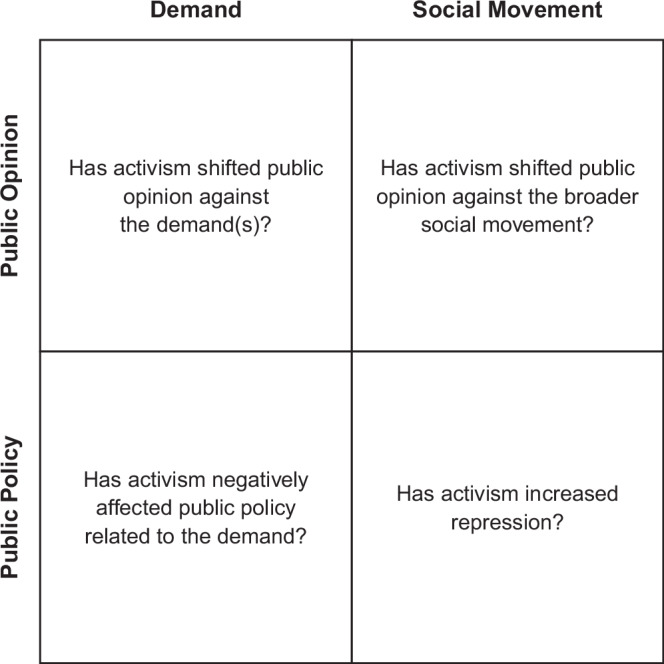
Framework for assessing counterproductivity. Two-by-two diagram considering the eff ects of social movements on public opinion and public policy relating to movement demand(s) and the movement itself.

In Fig. 1, we present our original two-dimensional framework for assessing counterproductivity, with Public Opinion and Public Policy on the vertical axis, and Demand versus Social Movement on the horizontal axis.

Operationalised to study JSO, this gives us the following questions:

A. Have JSO shifted public opinion against Net Zero policies?
B. Have JSO shifted public opinion against climate activism?
C. Have JSO negatively affected UK Net Zero policy?
D. Have JSO increased repression of protest in the UK?

The paper focusses primarily on A, but also addresses the other questions.

To research the impact of JSO, we conducted a rigorous mixed-methods study, analysing how JSO’s campaigning influenced media reporting and public opinion. This included media content analysis and original public opinion surveys to evaluate how JSO’s protests shaped discourse on climate policy, public attitudes, and government responses.

Our findings show that JSO achieved considerable media visibility. They had limited success in broadening public awareness of climate change, which was already very high. Their actions neither increased support for fossil fuel exploration nor diminished concern about climate change, challenging claims that their activism was counterproductive. However, they did contribute to increasing the salience of the issue of new oil and gas licences, compelling political actors to publicly address the matter.

Despite this visibility, JSO struggled to convert attention into broader influence. Their message was frequently diluted, with media coverage often framing them as disruptive, criminal, or a cultural flashpoint, rather than presenting their core demands, thereby limiting their ability to control the narrative.

This paper examines JSO’s impact and whether they have been counterproductive, as many of their critics claim^3^. The question of social movement effectiveness is widely debated across social science disciplines^9^, but remains difficult to assess^10^. As Nulman argues, ‘because […] competing claims are so difficult to assess, the impact of environmental movements remains highly contested’^11^. Movements rarely succeed or fail in isolation; rather, their influence emerges from a range of tactics and strategies used by different actors, including both institutional players and radical activists.

A hotly debated aspect of social movement impacts, particularly for small-scale civil disobedience groups like JSO, is the role of public opinion. Burstein went as far as to say that movements only influence policy through public opinion^12^. However, the relationship between public opinion and movement effectiveness is complex and bi-directional^13^. Time-series analyses of U.S. ecology, antinuclear, and peace movements suggest that protest activity rarely produces policy change in the absence of favourable public opinion and political allies, but can contribute to policy outcomes when embedded within such supportive contexts^14^. While strong public opinion on an issue can help social movements gain traction, this does not necessarily mean that movement activities themselves have shifted public attitudes. The causality may be reversed – public concern may lead to movement growth rather than the other way around.

One influential framework for assessing social movement impacts is Tilly’s WUNC model^15^, suggesting that movements gain effectiveness when they display worthiness, unity, numerical strength, and commitment. However, movements do not control how their WUNC attributes are interpreted; rather, the perception of these attributes is shaped by *discursive opportunity structures*^16^, that is, the media, political, and cultural environment. These structures shape the conditions under which movement claims gain legitimacy or are marginalised. Importantly, these structures are not static or neutral: they evolve over time and can be reinforced or contested by political actors, media practices, and policy debates. The role of climate obstructionist actors, seeking the opposite goals of JSO in shaping these structures has received increased attention^17^. Paterson et al. show how the UK’s net zero target, given its suggestion of a rapid transformation and association with technocratic elitism has functioned as a discursive opportunity for counter-movements to undermine and dismantle climate policy^18^. Similar trends have been observed in Italy^19^, and Germany^20^. As such, these structures are sites of political struggle, with media norms, dominant political narratives, and public sentiment shaping the extent to which a movement’s framing resonates or is distorted, thereby affecting perceptions of legitimacy, responsibility, and effectiveness^21^.

The study of media representation of protest has long been central to understanding movement outcomes, as public perception is often mediated through mainstream news coverage^22^. Media representation thus poses a significant challenge for social movements, as coverage of protest tends to be negative. Protest is frequently framed through the ‘protest paradigm’ ^23^, defined as ‘the routinized use of news frames, official sources, and public opinion to delegitimize protests and demonize protesters and maintain the status quo’ ^24^. Longitudinal research shows that activists’ ability to shape media coverage positively depends on building sympathetic journalist relationships and employing strategic framing, for example by leveraging celebrity endorsements^25^. A key variable in these studies is the extent to which activists are interviewed and able to communicate their messages directly to the public.

When looking specifically at the literature on disruptive protest, much of it has been concerned with questions of legitimacy and worthiness – which shape how movements are perceived and whether they can sustain public support. The civil disobedience literature has long debated when and how breaking the law is justified as a form of protest^26,27^. Recent debates have shifted towards the deliberative democratic qualities of civil disobedience^28,29^, questioning whether protest should be civil in nature and whether it constitutes a duty rather than just a right^30,31^. Recent work in political psychology complements this shift by arguing that symbolic, disruptive protest can interrupt motivated ignorance, forcing publics to confront issues they would otherwise seek to avoid^32^. Other literature has sought an ideal type of anarchist direct action, arguing that disruptive protest should neither be consequentialist, nor merely symbolic, but should be prefigurative – actively modelling the alternative social order it seeks to create^33,34^. The notion of ‘prefigurative legitimacy’^35^ suggests that a movement’s worthiness is reinforced when its tactics reflect its ideological commitments. From this perspective, disruptive protest is most effective when it embodies the values it seeks to promote rather than relying solely on rhetorical or instrumental strategies^36^.

Nevertheless, a key driver of the small-scale civil disobedience protests explored in this paper has been bold claims about the effectiveness of such protest, first by Extinction Rebellion^37^, and later by JSO and their A22 counterparts. Partly as a result, several scholars have explored the effectiveness of disruptive protest in particular. Some have pointed to the importance of disrupting elite economic interests^38^, making it more costly for decision-makers to resist activists’ demands than to concede^39^. However, disruptive protest carries significant risks. On the one hand, public support may decline^13^, contributing to what some call the ‘activist’s dilemma’ where disruptive protest increases visibility but alienates potential allies^6^. On the other hand, disruptive protest can invite repression^40^, as seen in several countries that have introduced stricter protest laws in response to climate activism^41^. Gunderson and Charles argued recently that based on existing literature, property destruction both reduced support for climate policy and enabled state repression^42^. Similar questions are important to interrogate the effects of disruptive non-violent protests. All this raises broader questions of counterproductivity—whether a movement’s tactics undermine its goals.

The so-called ‘radical flank effect’ provides another lens for understanding these dynamics. The main claim is that groups using more radical tactics may lose popularity while at the same time, support for more moderate organisations with similar demands increases^43^. This claim has received experimental support^44,45^ and has also been supported to a very small extent in surveys around real events^46^.

Overall, social movement impact and counterproductivity remains difficult to assess. Public opinion, media framing, and political responses interact in shaping outcomes. JSO’s case exemplifies these trade-offs: while increased visibility can compel political engagement, it may also lead to message dilution, unpopularity, and legal repression. This study builds on existing research by assessing JSO’s agenda-setting power, media representation, and political repercussions, contributing to debates on social movement impacts.

## Results and discussion

### Did JSO succeed in getting media attention?

**Fig. 2:**
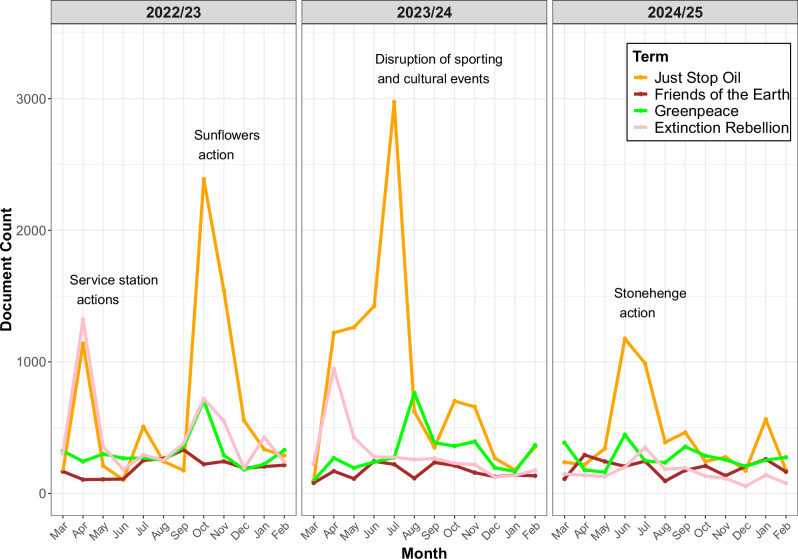
JSO mentions. Number of mentions of JSO in UK print media between March 2022 and March 2025, with mentions of Greenpeace, Friends of the Earth and XR plotted for comparison.

Figure 2 shows the number of UK media articles (per month) that mentioned “Just Stop Oil” from March 2022 until March 2025. Clear spikes in attention can be seen at various points, most notably in October 2022, when JSO activists threw soup at Van Gogh’s Sunflowers at the National Gallery in London, and July 2023, when there was a series of protest actions that targeted cultural and sporting events, including Wimbledon, the Ashes, the London Pride parade, and the BBC Proms. While most of the attention was focused on specific actions of the group, the trials and sentencing of its members also galvanised attention. For example, the sentencing of five JSO activists for periods of four to five years led to considerable media coverage in July 2024.

By way of comparison, Fig. 2 also shows the number of UK media articles (per month) that mentioned “Just Stop Oil”, “Greenpeace”, “Friends of the Earth” and “Extinction Rebellion” during the same time period. Greenpeace and Friends of the Earth are large NGOs that campaign actively on climate change in the UK. XR are a relevant point of comparison given that it has similar goals to JSO but has shifted its strategy away from a focus on disruptive actions. The figure indicates that JSO were successful in gaining considerably more attention than these other organisations/groups during the period under analysis. At its height, XR were the focus of intense attention – mentioned over 4000 times in UK print media during April 2019 – but lost momentum with the onset of the coronavirus pandemic, and have been declining in prominence since 2021. Figure 2 shows how JSO rapidly overshadowed XR; the latter group had a spike in coverage in April 2023, coinciding with “the Big One” march in London, but even this event, which attracted numbers that were two to three orders of magnitude greater than the largest JSO protest, was overshadowed by much smaller (disruptive) JSO actions that month.

### What was the nature of this attention?

Although JSO’s strategy of using high-profile disruptive direct action was clearly very successful in attracting media attention, they could not control the nature of this attention. The content of articles mentioning JSO frequently focused on the actions themselves (and the disruption to the public) rather than the demands of the campaign. We offer three forms of evidence of this focus: a) an objective coding of the subject categories of published articles, b) an analysis of the language used to describe JSO, and c) an analysis of the extent to which articles included direct quotes from the activists.

**Fig. 3:**
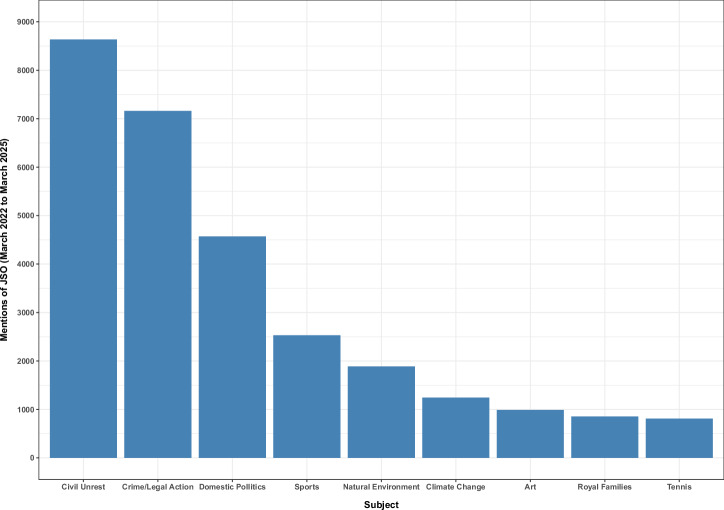
Newspaper subject categories. Subject categories of articles mentioning JSO between March 2022 and March 2025.

First, we consider the subject category of the published articles. Factiva categorises search results by “Subject”, using Subject categories such as “Climate Change”, “Domestic Politics”, etc.; this categorisation is based on an automated classification algorithm, though it may also be manually curated by editors. Figure 3 shows the major subject categories associated with articles mentioning JSO over the three-year period from March 2022 to March 2025.

As can be seen in the figure, the most commonly allocated category for JSO articles was “Civil Unrest”, followed by “Crime/Legal Action”. “Domestic Politics” was also a common category, but this largely reflected the political issue of how to respond to climate *protest*, as opposed to how to respond to climate change. As noted below, this category also reflected attempts by the government and right-leaning media to make a connection between JSO and the Labour Party. Over 2000 of the articles mentioning JSO were categorised as “Sport” (and indeed, the category of “Tennis” returned almost as many hits as “Climate Change”). This reflects JSO’s targeting of many major sporting events (cricket, tennis, rugby, football, golf, motor racing, darts, etc). This could conceivably be thought of as a success for the campaign: 427 articles categorised as Sports mentioned “climate change” in July 2023, which is by far the highest number in the last five years; the next two highest were the adjacent months, which probably also reflects JSO actions; by contrast, the mean number of mentions of climate change in Sports articles over the years 2021 through 2024 is 157. Thus, individuals who confine their newspaper reading to the sports pages are considerably more likely to have been exposed to the idea of climate change thanks to JSO actions. Some of the commentary may have addressed the issue seriously; for example, readers of the *Daily Mail* on 7 July 2023 may have encountered the reaction of football commentator Gary Lineker: ‘What is more important is probably our existence in the future rather than slight disruption of sporting events or other things. You don’t want things to be disrupted, but at the same time they will really be disrupted with climate change” ^47^. Nevertheless, the headline of this article – “Lineker defends the eco-clowns” – appears to be representative of the tone of most of the coverage of JSO’s actions at sports events, which tended to focus on disruption and was strongly critical of the protesters.

Indeed, the conjunction of “Just Stop Oil” with the use of terms like “eco-clowns” offers a second way to characterise the nature of the coverage. Among the wide range of pejorative neologisms in which the prefix “eco-” is combined with a term of derogation, e.g., “eco-yobs” (173 hits), “eco-clowns” (151), “eco-fanatics” (182), “eco-loons” (119), “eco-idiots” (123), etc., the most favoured by far was “eco-zealots”, which occurred in conjunction with JSO in 1353 articles between March 2023 and March 2025. This term was particularly favoured in the *Daily Mail*, where it was used in 792 of 2506 articles mentioning JSO in the period (32%). When Prime Minister Rishi Sunak used this term to describe JSO on 7 June 2023 it had already occurred in the *Daily Mail* 1068 times. In these articles “zealot” and “eco-zealot” were used as synonyms for “activist” and ”climate activist” respectively (e.g., “Just Stop Oil eco-zealots will be forced to pay compensation to the people whose lives they make a misery, under new plans being drawn up by Downing Street to combat protest mayhem”); the term was almost exclusively a descriptor that could be attributed to the author of the article or the subeditor who wrote the headline, with the only exceptions that we found being quotes attributed to Sunak.

The nature of the coverage in the *Daily Mail* is significant given that it is the newspaper with the highest paid circulation in the UK, and is also the newspaper that devoted the greatest attention to JSO, with over 2500 mentions in the studied period (the corresponding figures for the *Times*, the *Independent*, the *Telegraph* and the *Daily Express* were between 1300 and 1700). The *Daily Mail* is a right-leaning paper that has considerable influence over political discourse in the UK, particularly among Conservative Party voters and politicians^48^. It has a history of climate scepticism and continues to push against Net Zero policies. The general flavour of articles covering JSO in this outlet is captured by the fact that 31% of print articles mentioning JSO included at least one of the following terms: “arrogant”, “buffoons”, “clowns”, “crazy”, “eco-mob”, “eco-nut”, “extremist”, “fanatic”, “fools”, “imbecilic”, “idiot”, “loons”, “loopy”, “louts”, “mayhem”, “pathetic”, “retards”, “yobs”, “vandals”, “zealots”, with one of those terms occurring within five words of ”Just Stop Oil” in over 10% of all Daily Mail articles mentioning the group. Some caution is required here, given that conjunctive searches risk the possibility of misinterpretation, for instance if the author uses such terms ironically or critically (e.g., “These so-called ‘eco-zealots’ are making an important point”). However, we did not observe any examples like this in our reading of *Daily Mail* articles. Similarly, in a random sampling of 10% of the articles that did not use any of the pejorative terms listed above we found some articles that were relatively neutral (e.g., straightforward reports of trials), but did not observe any articles that included any positive or supportive comments about JSO.

It is also interesting to compare the paper’s treatment of JSO with its coverage during the same period of a campaign of protest against London’s Ultra Low Emission Zone (ULEZ) in which the cameras used to enforce the scheme were removed with electric saws. Individuals engaging in this destruction of public infrastructure are described as ”activists” or ”vigilantes”; an interview with one of them describes the ”masked maverick” as a father of two who is ”flouting the law to carry out what he calls ‘unpaid voluntary work’” (“*Moment masked ‘blade runner’ vigilante hacks London Ulez camera to the ground using electric saw in protest against Sadiq Khan’s hated £12.50-a-day driver tax*”, 28 August, 2024). Other articles about these anti-ULEZ protests quote positive reactions from members of the public (e.g., “These people are heroes, they appear to be the only ones fighting back”). The contrast indicates that the paper’s hostility towards JSO reflects less the disruptive or illegal nature of its actions than a broader delegitimisation of climate protest as such.

Although a similarly hostile attitude to JSO was evident in the *Daily Express*, the *Sun*, and the *Telegraph*, a more balanced tone was adopted in the *Guardian*, a left-leaning outlet. Of the fifteen articles in this newspaper in which one of the above pejorative terms appeared near a reference to JSO, almost all were quotes from politicians (Sunak or the deputy chairman of the Conservatives referring to “eco-zealots”, as well as Labour leader Keir Starmer and shadow chancellor Rachel Reeves referring to JSO as “pathetic”) or from *Daily Mail* headlines (“We’re shaping Labour policy boasts eco-mob”). There was also an opinion piece in which an ironic tone was adopted, in which the independent Climate Change Committee was compared to “those pesky ‘eco-zealots’ Just Stop Oil”. Indeed, over the three-year period the *Guardian* published several opinion pieces that were sympathetic to JSO, including pieces by regular columnist George Monbiot, Green party MP Caroline Lucas, and human rights lawyer Linda Lakhdir, as well as at least three pieces by members of JSO. There were also opinion pieces that were critical (e.g., one by Rupert Read referring to JSO’s tactics as “counterproductive” and another by Marina Hyde arguing that it was “wrong” for JSO to protest outside MP’s homes). Nevertheless, none of these critical opinion pieces could be described as especially condemnatory of JSO. Overall, it would be fair to describe the *Guardian’s* coverage as balanced and occasionally supportive.

**Fig. 4:**
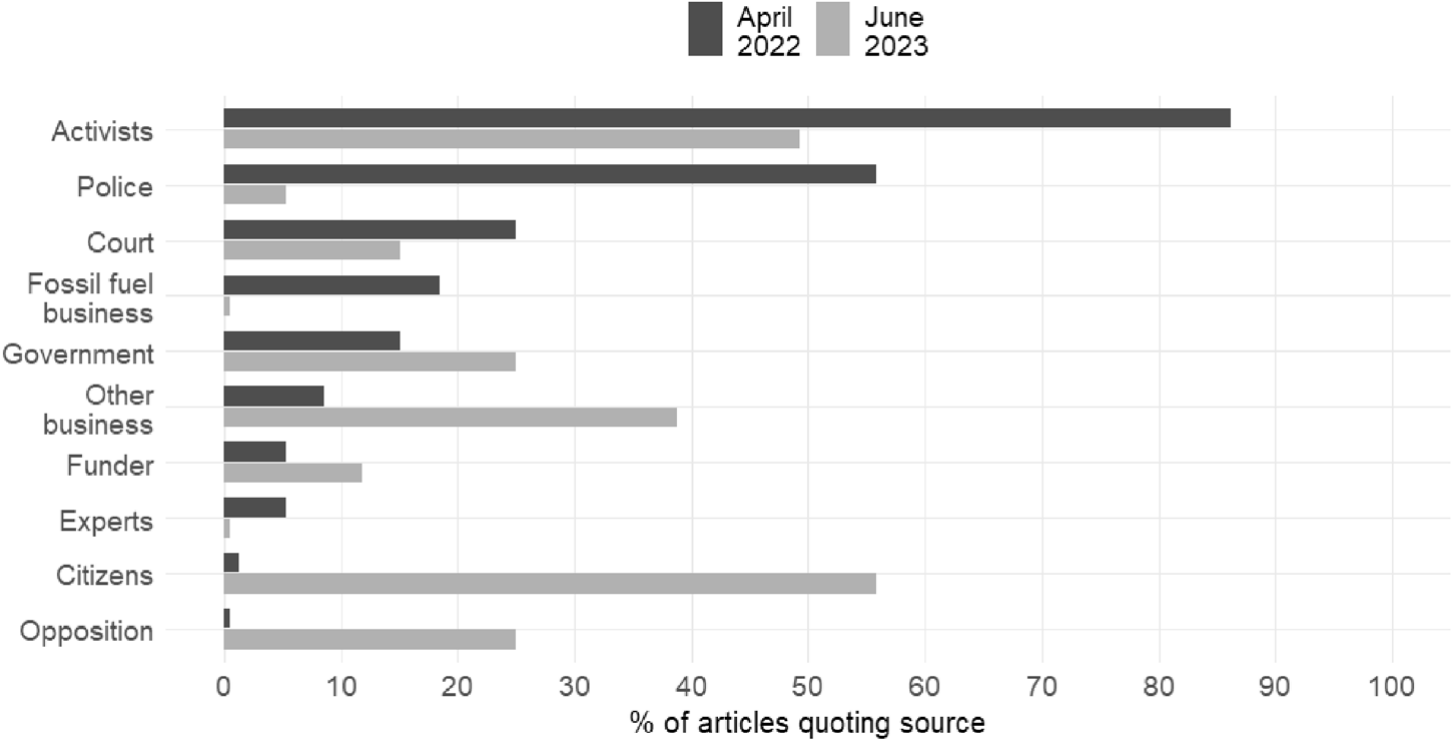
Quotes by type of actor. Percentage of random selection of articles mentioning JSO that quotes each type of actor.

As a third way of examining the nature of the coverage of JSO, we examined which sources were being quoted by reporters. In particular, we wished to test the possibility, suggested by our initial reading, that an initial framing that gave activists an opportunity to explain the rationale for their actions had given way to a dominant framing in which other actors expressed condemnation of the activists. We performed manual coding of a random selection of sixty articles mentioning JSO from all publications in April 2022 (representing its early emergence) and June 2023 (a high-salience period preceding the July 2023 wave). As can be seen in Fig. 4, in April 2022 the great majority of articles included quotes from activists, but by June 2023 the percentage had roughly halved. There was also a notable reduction in the number of articles that quoted police sources. By contrast, there was a large increase in the number of articles quoting citizens and affected businesses such as cultural institutions; these quotes were overwhelmingly negative. The other noteworthy difference between the two time points was the increase in the number of articles quoting government and opposition sources, reflecting the politicisation of JSO’s actions and demands.

In short, JSO were certainly effective in getting media attention and this media attention may have brought discussions of climate change to audiences that would not normally get to read about it. However, the bulk of reporting was very negative and dominated by ideologically hostile media outlets, with decreasing space for the JSO narrative in the reporting. Reporting was mainly in terms of unrest and law and order, rather than about climate change and climate policy. These findings confirm what protest paradigm research has told us, that demonisation is routinised and most frequently carried out by ideologically opposed news outlets^24,49^.

### Were JSO effective in communicating their message?

Notwithstanding the very negative tone of much of the JSO coverage, there is evidence suggesting that the group’s actions were successful in increasing coverage of the issue of new oil and gas licensing. Around 14% of the articles that mention JSO also made reference to oil and gas licences; that percentage fluctuated over time but was especially high in August 2023, when just over a third of the articles mentioning JSO also mentioned licences.

**Fig. 5:**
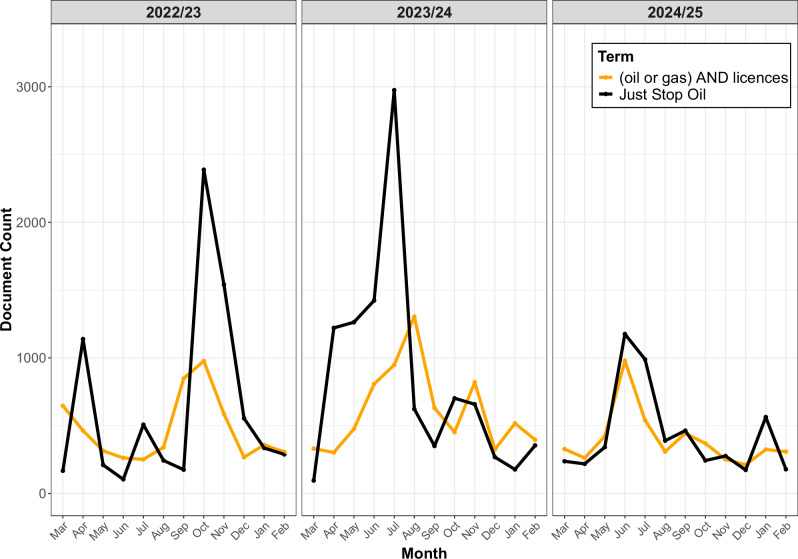
JSO and oil and gas licences mentions. Number of mentions of “Just Stop Oil” in UK print media between March 2022 and March 2025 was correlated with number of mentions of oil and gas licences.

Furthermore, even where the group’s demand was not explicitly reported they may nevertheless have influenced the policy agenda. As can be seen in Fig. 5, media attention to the issue of oil and gas licensing has varied over time, but appears to have tracked mentions of JSO. In particular, there was a marked increase in the number of articles that mentioned “oil” or “gas” and “licences” between March 2023 (106 articles) and August 2023 (1304 articles); this increase tracked (and followed) the spike in mentions of JSO over the same period. The two terms continued to exhibit a clear correlation in the months that followed. At times when JSO was relatively quiescent there was reduced coverage of oil and gas licensing, but this coverage increased when JSO hit the headlines again; in 2023 there was often a slight lag, such that licensing coverage followed the increase in JSO coverage. While this association does not demonstrate causality, the observed pattern is at least consistent with the possibility that JSO’s actions succeeded in drawing attention to the issue around which they were campaigning, in the same way in which Insulate Britain managed to increase attention to the issue of home insulation^50^. However, in months where there were extremely high numbers of mentions, JSO itself became the story, and there was not a corresponding increase in the coverage of oil and gas licences. At these times the protest paradigm became more salient, and negative coverage drowned out neutral and factual coverage. A similar shift over time was evident in left-leaning publications; for example, 19 of the 27 articles that mentioned JSO in the Guardian in April 2022 stated the group’s demand clearly (in addition to a small number that offered a slightly distorted version of the demand, e.g., ”an end to fossil fuels”), whereas only two of the 34 articles in June 2023 explicitly stated the demand, with most of these latter articles focusing on measures to suppress JSO’s actions, their links with Dale Vince and/or the Labour party, or the manner in which one of their activists had been removed from the field of play by an England cricketer.

To further explore how effective JSO were in communicating their message, we ran a small survey of the public in late July 2023, using Prolific (the sample for this survey was relatively diverse, but we do not claim that it constitutes a nationally-representative sample). Amongst our 250 respondents, 83% had heard of JSO. That is very high for a group of this size and obviously a result of the extensive media coverage that the group generated. However, only 17 out of 250 respondents in this non-representative sample gave a reasonably accurate account of JSO’s demand. That is, they specified that it was new oil or fossil fuel production that JSO was against, as opposed to just stopping all fossil fuel use immediately. This survey was carried out immediately after the then Secretary of State for Energy Security and Net Zero, Grant Shapps, had used JSO in his justification for renewing oil and gas licences.

In summary, JSO succeeded in attracting considerable media attention, in both absolute and relative terms. However, this attention was mostly negative. Though the left-leaning press was relatively balanced and even occasionally supportive (in its selection of opinion pieces), most media attention tended to focus on the group’s actions – their nuisance-value, immorality, selfishness, stupidity etc. – and what could/should be done about them, rather than on the group’s policy demand. Nevertheless, periods of high interest in JSO did correspond with more mentions of oil and gas licensing, and it seems unlikely that this was purely coincidental. Indeed, the government and their supporters actively sought to politicise JSO, linking them to the Labour Party, and using this to criticise the latter’s expressed policy of banning new oil and gas licences.

### Did JSO shift public opinion?

In assessing whether JSO’s media attention has led to a shift in public opinion, we need to look at public opinion on a variety of topics, including climate change concern, oil and gas licences, JSO themselves, and criminalisation of protest. These public opinion measurements are some key variables in the claim that JSO were counterproductive and address both the top-left and top-right squares in Fig. 1.

**Fig. 6:**
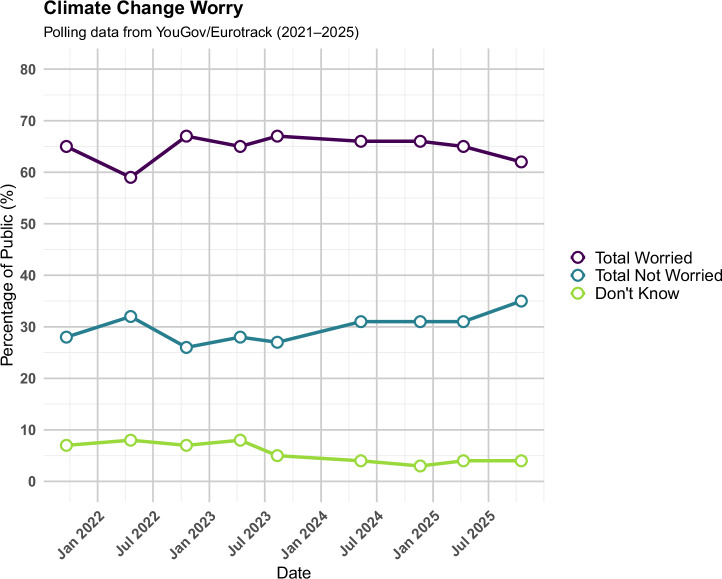
Tracking climate concern over time (2021–2025). This figure presents public responses to the question: “Thinking about how you feel TODAY about climate change and its effects, which of the following comes closest to your view?”

Public concern about climate change has remained consistently high in the UK for several years (see Fig. 6). This time period covers both the activity of Insulate Britain and that of JSO. In our commissioned survey^51^, 82% of respondents agreed that climate change is an important issue – including 74% of those who voted Conservative in 2019. This aligns with wider polling trends and underscores that JSO’s actions have not led to climate scepticism among the public. Nonetheless, the group itself remains highly unpopular. Only 16% of the public held a favourable view of JSO, a figure that masks significant political divergence: 33% of Labour voters viewed them favourably, compared to just 4% of Conservative voters. Another means of assessing whether JSO have shifted public opinion requires examining attitudes towards the issues they seek to influence.

**Fig. 7:**
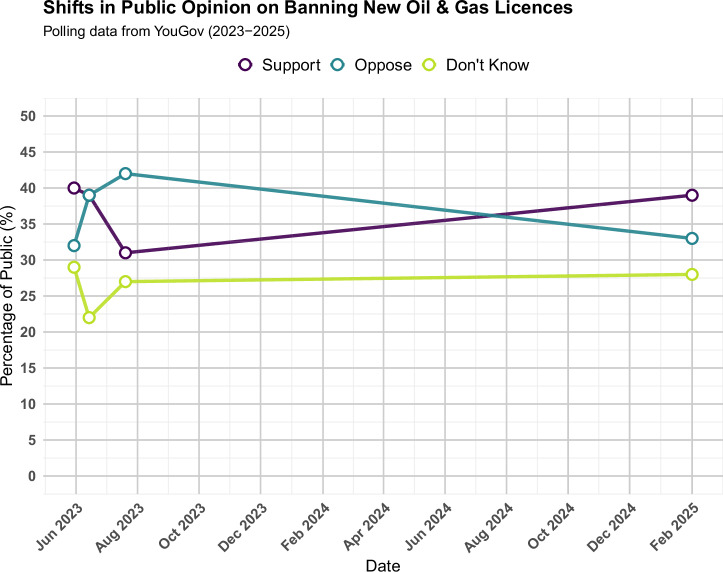
Tracking support for oil and gas licence ban over time (2023-2025). Public opinion on support versus opposition for banning new oil and gas licences.

JSO’s direct demand was to ban new oil and gas licences in the North Sea. In late spring and early summer 2023, the issue of renewing those licences became a major political topic, with the two main political parties taking opposing stances. In response, YouGov conducted surveys to gauge public opinion on the matter. Despite the media coverage of JSO protests, which correlated with an increase in mentions of oil and gas licences, JSO were unsuccessful in shifting public opinion toward supporting a ban on new licences. Public opinion, as shown in Fig. 7, shifted toward supporting oil and gas expansion instead.

In May 2023, more respondents supported rather than opposed a ban on new licences, but by June, support and opposition were equally split. By July 2023, opposition to a ban outnumbered support. The proportion of respondents choosing ‘Don’t Know’ fluctuated between 22% and 29%. It should be noted that women are much more likely to answer ‘Don’t Know’ in these surveys. For example, in our own survey, 40% of women answered ‘Don’t Know’, whilst only 17% of men did so^51^. Whilst we can only speculate about what drives this gendered divergence, it does somewhat limit the utility of issue-specific polling as a method of understanding politics and society in general. The trend of opinion shifting towards opposing a ban is clearly reflected in the graph, and corroborating evidence comes from another YouGov poll conducted on July 31, 2023^52^. While the phrasing of the poll differed – asking whether respondents thought it was “right or wrong” for the government to issue new licences – the results still aligned with the broader trend. This poll found that more respondents thought it was “right” to issue new licences (42% vs. 27%), with 31% selecting “Don’t Know,” supporting the shift toward greater opposition to a ban. By February 2025, this trend had reversed, with support for a ban on new licences outpacing opposition, echoing proportions similar to those seen in May 2023.

This movement in public opinion was largely driven by Conservative voters from the 2019 General Election, who showed the most significant shift in their stance. In contrast, Labour and Liberal Democrat voters showed a slight shift in favour of supporting a ban on new oil and gas licences. JSO were a key element of the political discourse that influenced this shift. The Conservative government explicitly used JSO as a justification for renewing licences, framing it as essential for the UK’s energy security and sovereignty by reducing dependence on imports, particularly from Russia in light of their invasion of Ukraine^53^. Energy Security Secretary Grant Shapps described not renewing the licences as ‘bonkers’ and referred to the Labour Party as the ‘political wing of Just Stop Oil’ ^54^. The polling shows that this discourse may have been successful in shoring up support for the policy decision. This was one of several policy areas in which the UK government decreased its commitment to Net Zero policies at the time. Whilst JSO were used in the political discourse, it is unlikely that it was JSO that shifted opinion towards opposing a ban in 2023. After all, as our Prolific survey showed, the vast majority of people were unaware that a ban on new licences was JSO’s demand. Since it was Conservative voters who shifted opinion, it is much more likely that they were influenced by Government discourse, not least around energy independence from Russia.

Moving to the bottom-right square in Fig. 1, the high profile of JSO meant that they became central to the political discourse surrounding the criminalisation of protest. Their tactics have been invoked explicitly in parliamentary debate and media commentary as justification for expanding the powers of the police and courts. Another way to assess the group’s broader impact is to assess how it may have influenced, or been caught up in, changing public attitudes toward the policing and sentencing of protest actions more broadly. Public opinion on the criminalisation of protest had already begun shifting prior to JSO’s emergence. Support for tougher protest laws was evident even before JSO came to public prominence. A YouGov survey from February 2022 – prior to the group’s emergence – found that 35% of the public thought policing of protest was “not strict enough,” 30% thought it was “about right,” and only 16% felt it was “too strict” with 18% selecting “Don’t know.” At the same time, there was majority support, 63%, for provisions in the (then-proposed) Police, Crime, Sentencing and Courts Bill that would criminalise tactics like road-blocking and slow marches^55^. This created the impression of a public largely aligned with the government’s increasingly punitive stance on protest.

However, our 2023 survey – conducted after the legislation had come into effect and following a year of sustained JSO activity – suggests a more complex picture^51^. The public does not appear to support the severity of penalties now enshrined in law. This question was purposely asked before the question about JSO, in order not to create the connection in respondents’ minds between JSO and criminalisation of protest. When asked what punishment was most appropriate for “non-violent but disruptive protest, such as blocking a road”, only 29% supported prison sentences. By contrast, 39% favoured no punishment or only a minor one, and 25% preferred alternatives such as large fines or community service. Whilst our research shows that there was little appetite in 2023 for criminalising protest amongst the public; media and policymakers have nonetheless used JSO as a reason for such criminalisation. A 2024 survey referring specifically to sentences of JSO activists shows that a majority of the public welcomed the harsh sentences handed out^56^. People are then much more likely to support criminalisation of protest when it refers specifically to JSO.

## Discussion

This paper has asked to what extent smaller-scale civil disobedience campaigns have an impact on achieving political outcomes. In doing so, it has taken seriously the allegation that such campaigns may be counterproductive. To assess counterproductivity, we set out a two-dimensional framework in Fig. 1. This is a significant contribution to interdisciplinary academic and public debate that will greatly improve understanding of counterproductivity. The case we studied has been one of the most prominent such campaigns in the post-pandemic context, namely the British group Just Stop Oil. Through rigorous media content analysis and public opinion surveys, we have sought to capture the effects of JSO’s campaign. We did so through addressing four sub-questions concerning media attention, communication of the message, and effects on public opinion.

In answer to our first question, the media content analysis showed us that JSO were remarkably successful in gaining media attention. This led to very high levels of awareness of the group. However, in response to our second question, the fact that the bulk of this media attention has been hostile and printed in outlets that are ideologically opposed to JSO, has no doubt contributed to JSO’s unpopularity.

In relation to the third question, about effectiveness of communicating its message, the answer is more ambiguous. Only 14% of media coverage mentioned oil and gas licences, and a mere 7% of respondents correctly identified JSO’s main demand, suggesting limited effectiveness in communicating the message. This is possibly caused by the decreasing space given to JSO activists to speak for themselves in media reporting, rather than being reported about. Taken together, this indicates low effectiveness in communicating JSO’s core message. However, there is a strong correlation between reporting about JSO and reporting about oil and gas licences. It seems therefore that JSO’s protest contributed to increasing the salience of the issue of oil and gas licences. Both the Conservative government and the opposition Labour party took public stances for and against new licences, and the former invoked JSO in doing so. We can therefore say that JSO contributed to politicising and raising the salience of the issue.

Our last question, on public opinion, gets to the heart of questions about whether JSO’s unpopularity means that it was counterproductive. That is, if we were to see shifts away from concern about climate change, or towards endorsing new oil and gas licences amongst the public, that would be counterproductive. We can, however, see very little movement in concern about climate change. Nor have JSO directly affected public opinion on North Sea oil and gas licences. It was likely Government discourse that temporarily shifted opinion in favour of further oil and gas exploration, but public opinion on that matter has then returned to roughly where it was prior to the issue being highly salient.

In brief, JSO succeeded in getting media attention, contributed to raising the salience of oil and gas licences in the North Sea, and politicising the issue. They had no discernible effect on public opinion about climate change or oil and gas licences, and whether any political parties would have taken different stances without JSO campaigning is impossible to say. In that sense, they have been neither effective nor counterproductive.

JSO’s case aligns with the protest paradigm literature^24,49^. Their message has been shaped by discursive media constraints that favour spectacle over substance. While JSO successfully gained media visibility, their core demand of ending new fossil fuel licences struggled to gain traction, as reporting often emphasised disruption, criminality, and public disapproval over policy concerns. This reinforces the challenge of message dilution – a phenomenon in which a movement’s visibility is high, but its ability to define the narrative is weak. If sympathetic journalists are something that can help movements mitigate the protest paradigm^25^, sympathetic reporting has been drowned out by reporting in hostile outlets. Importantly, other research has shown how organised political actors have successfully characterised climate mitigation polices as economically harmful, elitist, or disconnected from everyday concerns^18–20^. Within such a context, disruptive protest is more easily interpreted through frames of irresponsibility or extremism, further undermining JSO’s efforts to draw attention to the core demands. The extent to which the responsibility for this framing lies with JSO should not be exaggerated.

The WUNC model^15^ helps explain how hostile media reporting has been successful in demonising JSO. The movement exhibits strong unity and commitment, but the media has successfully painted them as unworthy. The necessarily small number of activists have helped this endeavour. Their small activist base and controversial tactics have made it easier for opponents to frame them as fringe rather than representative of a broader climate movement. This dynamic reinforces the difficulty of securing broad-based public support, even when a movement’s core demand aligns with widespread climate concerns. JSO have then been limited by the available discursive opportunity structures^16^, which have been heavily influenced by climate obstructors^17^.

The main focus of this paper has been on the top-left square of Fig. 1, where the evidence shows that JSO have not been counterproductive in terms of public sentiment about Net Zero policies. Regarding the bottom-left square, although the previous Conservative Government sought to link JSO with Labour, the current UK Labour Government has continued to pursue its Net Zero policies, including the ban on new North Sea oil and gas licences. This indicates that JSO were also not counterproductive in relation to Net Zero public policies. The top-right square, concerning the broader climate movement, is less directly addressed here. While it is clear that the vast majority of the public dislike JSO, the implications of this for the wider movement remain uncertain. Radical Flank Effect scholarship is more directly concerned with this question, and the jury is still out.

Lastly, in relation to the bottom-right square, JSO’s actions were cited by the Conservative Government in justifying anti-protest laws, which, according to police statistics, were primarily applied to JSO^57^. These anti-protest laws are also part of a global trend toward criminalising protest^41^. It would be unfair to pin the blame for this repression on those being repressed, even though JSO’s media-manufactured unpopularity was instrumentalised by the preceding Government to justify repression. In this sense, JSO may have been ‘possibly’ or ‘somewhat’ counterproductive in contributing to repressive public policy against climate and other types of activism – but importantly, this is not the type of counterproductivity that is typically referenced by critics of disruptive protest. To summarise, JSO have not been counterproductive in turning the public or policymakers against Net Zero policies. Their impact on broader climate movement support and state repression of protest is more ambiguous.

## Methods

This paper assesses the impact of JSO through four research questions:

1. Did JSO succeed in getting media attention?
2. What was the nature of this attention?
3. Were JSO effective in communicating their message?
4. Did JSO shift public opinion?

Assessing the impact of smaller-scale civil disobedience campaigns like JSO requires a multi-method approach, as the influence of protest movements is often contested and difficult to isolate from broader political and societal trends. This study focuses on public opinion, media representation, and agenda-setting effects to assess the extent to which JSO’s actions shaped discourse around climate policy and protest legitimacy.

### Data sources and methods

We adopted a triangulation strategy, combining media content analysis and public opinion surveys to examine:

1. General public concern about climate change
2. Public opinion on banning new oil and gas licences (JSO’s primary policy demand)
3. The (un)popularity of JSO and civil disobedience
4. Public opinion on the criminalisation of protest
5. Media reporting on JSO

### Media content analysis

We used the Factiva database to track mentions of “Just Stop Oil” and related search terms in UK print media from March 2022 (when JSO announced itself to the world) through to March 2025 (when it announced it would cease further public actions). To examine media framing and the extent to which JSO’s demands were communicated, we manually coded 60 articles published in April 2022 and June 2023, capturing sources quoted and the length of quotes. These months were selected to represent periods of peak activity.

### Polling data

YouGov intermittently surveyed questions relevant to this project, including support for banning new oil and gas licences and the popularity of civil disobedience groups. To explore how these factors relate to each other, it was important to ask all questions of the same respondents rather than separately. Accordingly, we designed a survey with five questions covering:

1. Top issues facing the country: Respondents were asked to pick their top three issues from 14 options, including “The Environment,” following the standard YouGov formulation.
2. Support for banning new oil and gas developments in the North Sea (JSO’s main policy demand).
3. Opinions on punishment for non-violent disruptive protest.
4. Views of JSO.
5. Perceived importance of climate change

We also compiled longitudinal YouGov public opinion data from 2021 to 2025. As these surveys were not centrally archived, we manually collected data from published sources. We then created summary figures showing changes in public concern about climate change and support for JSO’s policy demands over time. Full details of the data sources and the code used to generate the figures are available on the Open Science Framework.

### Prolific survey

We used Prolific Academic to ask a different set of non-representative respondents if they had heard of JSO and if they knew what their demands were.

## Data Availability

The data are available here: https://osf.io/r2qhn/files/osfstorage.

## References

[1] Kinyon, L., Dolšak, N. & Prakash, A. When, where, and which climate activists have vandalized museums. *npj Clim. Action* **2**, 27 (2023).

[2] Araya López, A. & Davis, C. J. On art and the limits of dissent: climate activism at museums and galleries. *Protest* **4**, 143–176 (2024).

[3] Mann, M. E. Throwing soup at art shifted people’s views of climate protests—but maybe not in the right way. upenn.edu https://web.sas.upenn.edu/pcssm/news/throwing-soup-at-art-shifted-peoples-views-of-climate-protests-but-maybe-not-in-the-right-way/ (2022).

[4] Phillips, A. *Energy Minister Blasts Insulate Britain for ‘Standing in the Way’ of Progress* (Daily Express, 2022).

[5] Thomas, E. F. & Louis, W. R. When will collective action be effective? violent and non-violent protests differentially influence perceptions of legitimacy and efficacy among sympathizers. *Pers. Soc. Psychol. Bull.* **40**, 263–276 (2014). 24311435 10.1177/0146167213510525

[6] Feinberg, M., Willer, R. & Kovacheff, C. The activist’s dilemma: extreme protest actions reduce popular support for social movements. *J. Pers. Soc. Psychol.* **119**, 1086–1111 (2020). 31928025 10.1037/pspi0000230

[7] Verkuyten, M., Adelman, L. & Yogeeswaran, K. Intolerance of transgressive protest actions: the differential roles of deontological and utilitarian morality. *Pers. Soc. Psychol. Bull.* **49**, 1184–1196 (2023). 35638641 10.1177/01461672221099709 PMC10320703

[8] JSO. Just Stop Oil. https://juststopoil.org/ (2024).

[9] Fisher, D. R., Berglund, O. & Davis, C. J. How effective are climate protests at swaying policy — and what could make a difference? *Nature* **623**, 910–913 (2023). 38017266 10.1038/d41586-023-03721-z

[10] Uba, K. Research methods for studying collective action outcomes. In *Handbook of Research Methods and Applications for Social Movements* (eds Cox, L., Szolucha, A., Arribas Lozano, A. & Chattopadhyay, S.) 420–431 (Edward Elgar, 2024).

[11] Nulman, E. The impacts of environmental movements. In *The Oxford Handbook of Social Movements* (eds Della Porta, D. & Diani, M.) 729–742 (Oxford University Press, 2015).

[12] Burstein, P. Social movements and public policy. In *How Social Movements Matter* (eds Giugni, M., McAdam, D. & Tilly, C.) 3–21 (University of Minnesota Press, 1999).

[13] Uba, K. Political protest and policy change: the direct impacts of indian anti-privatization mobilizations, 1990-2003. *Mobilization* **10**, 383–396 (2005).

[14] Giugni, M. Useless protest? A time-series analysis of the policy outcomes of ecology, antinuclear, and peace movements in the United States, 1977-1995. *Mobilization* **12**, 53–77 (2007).

[15] Tilly, C. *Social Movements, 1768–2004* (Routledge, 2019). 10.4324/9781315632063.

[16] McCammon, H. Discursive opportunity structure. In *The**Wiley-Blackwell Encyclopedia of Social and Political Movements* (Wiley, 2013). 10.1002/9780470674871.wbespm073.

[17] Brulle, R. J., Roberts, J. T. & Spencer, M. C. *Climate Obstruction across Europe* (Oxford University Press, 2024).

[18] Paterson, M., Wilshire, S. & Tobin, P. The rise of anti-net zero populism in the UK: comparing rhetorical strategies for climate policy dismantling. *J. Comparative Policy Anal.* **26**, 332–350 (2024).

[19] Colantone, I., Di Lonardo, L., Margalit, Y. & Percoco, M. The political consequences of green policies: evidence from Italy. *Am. Political Sci. Rev.* **118**, 108–126 (2024).

[20] Grünwald, L. & Patterson, J. Roadblocks of polarization: Interpretive mechanisms of opposition to a speed limit policy on German highways. *Energy Res. Soc. Sci.* **122**, 104009 (2025).

[21] Meyer, D. S. & Staggenborg, S. Movements, countermovements, and the structure of political opportunity. *Am. J. Sociol.* **101**, https://about.jstor.org/terms (1996).

[22] McLeod, D. M. & Hertog, J. K. The manufacture of ‘public opinion’ by reporters: informal cues for public perceptions of protest groups. *Discourse Soc.* **3**, 259–275 (1992).

[23] Chan, J. M. & Lee, C.-C. The journalistic paradigm on civil protests: a case study of Hong Kong. In *The New Media in National and International Conflict* (eds, Arno, A. & Dissanayake, W.) 183–202 (Westview, Boulder, 1984).

[24] Harlow, S. & Brown, D. K. A new protest paradigm: toward a critical approach to protest news analyses. *Int. J. Press Polit.* **28**, 333–343 (2023).

[25] Rauch, J. et al. From Seattle 1999 to New York 2004: a longitudinal analysis of journalistic framing of the movement for democratic globalization. *Soc. Mov. Stud.* **6**, 131–145 (2007).

[26] Brownlee, K. *Conscience and Conviction* (Oxford University Press, 2012).

[27] Rawls, J. *A Theory of Justice* (Harvard University Press, 1971).

[28] Celikates, R. Democratizing civil disobedience. *Philos. Soc. Crit.* **42**, 982–994 (2015).

[29] Smith, W. Deliberative democratic disobedience. In *The Cambridge Companion to Civil Disobedience* (ed. Scheuerman, W. E.) 105–127 (Cambridge University Press, Cambridge, 2021).

[30] Delmas, C. *A Duty to Resist: When Disobedience Should Be Uncivil*. (Oxford University Press, 2018).

[31] Scheuerman, W. E. Political disobedience and the climate emergency. *Philos. Soc. Crit.* **48**, 791–812 (2022).

[32] Hayward, C. R. Disruption: what is it good for? *J. Polit.* **82**, 448–459 (2020).

[33] Franks, B. The direct action ethic. *Anarchist Stud.* **11**, 16–41 (2003).

[34] Graeber, D. *Direct Action: An Ethnography* (AK Press, Edinburgh, 2009).

[35] Berglund, O. Disruptive protest, civil disobedience & direct action. *Politics* 026339572311769, 10.1177/02633957231176999 (2023).

[36] Badullovich, N. et al. How does public perception of climate protest influence support for climate action? *npj Clim. Action* **3**, 16 (2024).

[37] Berglund, O. & Schmidt, D. *Extinction Rebellion and Climate Change Activism* (Palgrave Pivot, London, 2020).

[38] Young, K. A. & Thomas-Walters, L. What the climate movement’s debate about disruption gets wrong. *Humanit. Soc. Sci. Commun.* **11**, 25 (2024).

[39] Bailey, D. J. Resistance is futile? The impact of disruptive protest in the ‘silver age of permanent austerity. *Socioecon Rev* **13**, 1–34 (2014).

[40] Wang, D. J. & Piazza, A. The use of disruptive tactics in protest as a trade-off: the role of social movement claims. *Social Forces* **94**, 1675–1710 (2016).

[41] Rossdale, C., Berglund, O., Pantazis, C., Pessoa Cavalcanti, R. & Franco Brotto, T. The global criminalisation and repression of climate and environmental protest – a repertoire of repression. *Environ. Polit.* 1–26, 10.1080/09644016.2025.2602416 (2025).

[42] Gunderson, R. & Charles, W. A sociology of “climatage”: the appeal and counterproductivity of property destruction as a climate change strategy. *Environ. Sociol.* **9**, 398–408 (2023).

[43] Chamberlain, M. C. R. & Madsen, O. J. Rebels with a cause: public attitudes on radical protest actions a review of empirical evidence of radical flank effects. *Human Arenas*10.1007/s42087-025-00485-y (2025).

[44] Dasch, S. T., Bellm, M., Shuman, E. & van Zomeren, M. The radical flank: curse or blessing of a social movement? *Global Environ. Psychol.* **2**, e11121 (2024).

[45] Simpson, B., Willer, R. & Feinberg, M. Radical flanks of social movements can increase support for moderate factions. *PNAS Nexus* **1**, 1–11 (2022). 10.1093/pnasnexus/pgac110 PMC9896934 36741469

[46] Ostarek, M., Simpson, B., Rogers, C. & Ozden, J. Radical climate protests linked to increases in public support for moderate organizations. *Nat. Sustain.* **7**, 1626–1632 (2024).

[47] Matthews, C. Gary Lineker is Slammed as a ‘Hypocrite in the Highest Order’ for Defending Just Stop Oil Protesters After Eco-clowns Stormed Wimbledon Court and Threw Jigsaw Pieces. Daily Mail https://www.dailymail.co.uk/news/article-12273279/Gary-Lineker-slammed-hypocrite-highest-order-defending-Just-Stop-Oil.html (2023).

[48] Stoegner, K. & Wodak, R. ‘The man who hated Britain’ – the discursive construction of ‘national unity’ in the Daily Mail. *Critical Discourse Stud.* **13**, 193–209 (2016).

[49] Kim, K. & Shahin, S. Ideological parallelism: toward a transnational understanding of the protest paradigm. *Soc Mov Stud* **19**, 391–407 (2020).

[50] Davis, C. *Just Stop Oil: Do Radical Protests Turn the Public Away from a Cause? Here’s the Evidence* (The Conversation, 2022).

[51] YouGov. YouGov University of Bristol Survey 1 August 2023. yougov.co.uk https://d3nkl3psvxxpe9.cloudfront.net/documents/UniversityofBristol_Results_230720_W.pdf (2023).

[52] YouGov. YouGov North Sea Survey 31 July 2023. yougov.co.uk https://yougov.co.uk/topics/politics/survey-results/daily/2023/07/31/aac1f/1 (2023).

[53] Kuzemko, C., Blondeel, M., Dupont, C. & Brisbois, M. C. Russia’s war on Ukraine, European energy policy responses & implications for sustainable transformations. *Energy Res Soc Sci* **93**, 102842 (2022).

[54] City A. M. Shapps: Labour ‘sponsored by Just Stop Oil’ in light of ‘bonkers’ fossil fuel project ban. City A.M. https://www.cityam.com/shapps-labour-sponsored-by-just-stop-oil-in-light-of-bonkers-fossil-fuel-project-ban/ (2023).

[55] YouGov. Policing Bill: Britons support proposed new police protest powers. yougov.co.uk https://yougov.co.uk/politics/articles/41561-policing-bill-britons-support-proposed-new-police- (2022).

[56] YouGov. Most Britons say Just Stop Oil protestors deserved jail time. yougov.co.uk https://yougov.co.uk/politics/articles/50766-most-britons-say-just-stop-oil-protestors-deserved-jail-time (2024).

[57] Home Office. Police protest powers, June 2022 to March 2024. gov.uk https://www.gov.uk/government/statistics/police-protest-powers-june-2022-to-march-2024/police-protest-powers-june-2022-to-march-2024 (2024).

